# A novel deep learning framework with dynamic tokenization for identifying chromatin interactions along with motif importance investigation

**DOI:** 10.1093/bib/bbaf289

**Published:** 2025-06-19

**Authors:** Liangcan Li, Xin Li, Hao Wu

**Affiliations:** School of Software, Shandong University, No. 1500, Shunshun Street, High tech Zone, Jinan, Shandong 250100, China; School of Software, Shandong University, No. 1500, Shunshun Street, High tech Zone, Jinan, Shandong 250100, China; School of Software, Shandong University, No. 1500, Shunshun Street, High tech Zone, Jinan, Shandong 250100, China; Shenzhen Research Institute of Shandong University, Shenzhen 518063, Guangdong, China

**Keywords:** chromatin interactions, deep learning framework, dynamic tokenization, word embedding, motif importance

## Abstract

A comprehensive understanding of chromatin interaction networks is crucial for unraveling the regulatory mechanisms of gene expression. While various computational methods have been developed to predict chromatin interactions and address the limitations and high costs of high-throughput experimental techniques, their performance is often overestimated due to the specificity of chromatin interaction data. In this study, we proposed Inter-Chrom, a novel deep learning model integrating dynamic tokenization, DNABERT’s word embedding, and the efficient channel attention mechanism to identify chromatin interactions using sequence and genomic features, leveraging a newly curated dataset. Experimental results demonstrate that Inter-Chrom outperforms existing methods on three cell line datasets. Additionally, we proposed a novel method for calculating motif importance and analyzed the motifs with high importance scores identified through this method, including those that have been extensively studied and others that have received limited attention to date. Inter-Chrom’s robustness for input variations and superior ability to leverage sequence features position it as a powerful tool for advancing chromatin interaction research. The source code of Inter-Chrom is freely available at https://github.com/HaoWuLab-Bioinformatics/Inter-Chrom.

## Introduction

The three-dimensional (3D) organization of the genome plays a pivotal role in regulating cellular functions and pathological processes, particularly through chromatin interactions [1, 2]. These interactions are crucial for gene transcription, regulation, and expression, as they modulate the accessibility of regulatory elements to target genes, thereby affecting transcriptional efficiency [3–10]. By synergizing with specific gene promoters, activating transcription, and facilitating enhancer-promoter communication, chromatin interactions serve as integral mechanisms for enhancing gene expression [11–17].

Recent advancements in genomic technologies, such as high-throughput chromosome conformation capture (Hi-C) [18] and chromatin interaction analysis with paired-end tags (ChIA-PET) [19], have substantially expanded our knowledge of genome-wide chromatin architecture [20–22]. FitHiC2 [23] provides a computational foundation for large-scale interaction analysis through statistical models. Peakachu [24] framework improves detection sensitivity and achieves cross-platform prediction through supervised learning. cLoops2 [25] provides an integrated solution as a full-stack tool, jointly promoting research towards machine learning driven and multi-omics integration. While these technologies provide detailed mapping of chromatin architecture, their high costs limit broader applications. To overcome this challenge, various computational methods have been developed to efficiently predict chromatin interactions at lower costs. Prominent tools such as TargetFinder [26], JEME [27], and RIPPLE [28] leverage multiple functional genomic datasets, including open chromatin data, transcription profiles, and chromatin immunoprecipitation sequencing (ChIP-seq) data for transcription factors and histone modifications. In addition, sequence-based approaches like SPEID [29], PEP [30], and EPIsHilbert [31] have made some breakthroughs and progress in identifying chromatin interactions.

Despite these advancements, predicting chromatin interactions remains challenging due to the inherent complexity and overlapping nature of genomic datasets. Traditional random sampling methods often lead to model overfitting, which could result in overestimated model performance [32–34]. To address this, Whalen et al. introduced a chromatin interaction dataset alongside a chromosome-split strategy for training and prediction. However, their models underperformed, and the evaluation metrics used were not sufficiently comprehensive in assessing the model’s effectiveness. In contrast, IChrom-Deep [35] introduced an attention mechanism-based module to improve sequence feature extraction and combine genomic features, significantly advancing chromatin interaction prediction. However, its sequence feature extraction capabilities still leave room for improvement.

To address these challenges of chromatin interaction prediction, we propose Inter-Chrom, a novel deep learning framework based on dynamic tokenization. Inter-Chrom is designed to extract sequence features efficiently and utilizes four evaluation metrics to assess performance comprehensively on a balanced test set. The primary contributions of this study are as follows: (i) Novel sequence generalization strategy: We introduce a method to extract top-k words based on length and frequency for both forward and reverse strands, enhancing sequence information utilization. (ii) Advanced deep learning framework: Inter-Chrom combines dynamic tokenization with DNABERT’s word embedding for robust chromatin interaction prediction. (iii) Superior model performance: Our model consistently outperforms existing approaches in chromatin interaction prediction across multiple metrics. (iv) Chromosome-split training strategy: We emphasize the importance of avoiding sequence data leakage and analyze the impact of varying sample sizes on model performance. (v) Motif influence analysis: We assess the effect of individual motifs, analyze overlapping motif contributions, and propose a novel solution to address these complexities. (vi) New motif importance computation method: Our approach overcomes the limitations of previous methods, providing a more comprehensive perspective on motif influence. (vii) High-ranking motifs: We identify and highlight key motifs with significant roles in chromatin interaction prediction.

## Materials and methods

### Datasets

In this study, we adopted the benchmark dataset provided by TargetFinder2.0 [36] as the basis for our analysis. This dataset encompasses four distinct human cell lines, namely K562, HeLa-S3, IMR90, and GM12878. Each sample within the dataset is enriched with a diverse set of genomic features, including k-mer frequencies, genomic coordinates of chromatin bins, sequence conservation scores, CTCF binding motifs, and distances between chromatin bins.

To ensure balanced representation, we implemented a strict filtering process based on chromatin states, maintaining a ratio of 1:10 between positive samples (with interactions) and negative samples (without interactions) for each cell line. However, due to the limited number of positive samples in the HeLa-S3 cell line (only 66 after filtration), we excluded this cell line from further analysis. For the remaining cell lines, sequence data was extracted based on genomic coordinates using BEDTools [37]. Detailed information on the dataset used in this study is provided in Table 1.

**Table 1:** **The details of the datasets.** This table provides the number of positive and negative samples, the positive-to-negative sample ratio, and the genomic features available for each cell line.

**Cell lines**	**Positive samples**	**Negative samples**	**Genomic features**
K562	857	8570	1139
IMR90	2148	21480	129
GM12878	2789	27890	459

### Model architecture

**Figure 1:**
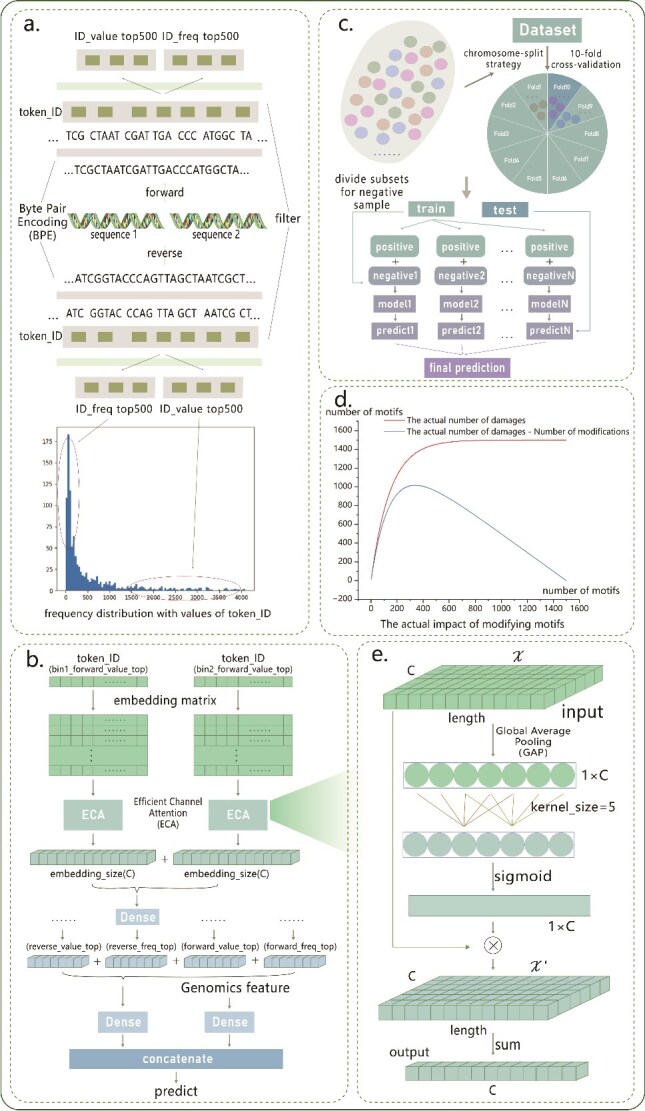
Model architecture and training procedure of Inter-Chrom. (a). Preprocessing of sequence data. Each sample contains two DNA sequences. Taking one sequence as an example, the original sequence is first processed in both forward and reverse directions to generate two sequences, which are then divided into a set of different short sequences. Based on predefined word length and frequency criteria, the top 500 most frequent words are selected to construct standardized input sequences for model training. (b). The model architecture of Inter-Chrom. Inter-Chrom consists of a sequence module and a genome module. The sequence module takes the preprocessed result from (a) as input, and the genome module uses genome features, conservation scores, CTCF motifs, and the distance between chromatin bins as input. (c). Training process. The training process addresses an imbalance between positive and negative samples in the training set. The final prediction is obtained by averaging the outputs of multiple sub-models. (d). Impact of motif erasure. This panel depicts the number of motifs affected after erasing varying amounts of specified motif information in the DNA sequence. (e). Detailed process of the ECA module within the framework. This panel illustrates the specific steps involved in the ECA module.

Inter-Chrom is a deep learning model with efficient channel attention mechanisms designed to integrate DNA sequences and genomic features for chromatin interaction prediction. The architecture, illustrated in Fig. 1, comprises two primary components: a sequence module and a genomic module.

In the sequence module, SentencePiece and Byte Pair Encoding (BPE) are employed for tokenizing DNA sequences. SentencePiece is a language-agnostic tokenizer that processes each input as a continuous stream of raw data, bypassing predefined tokenization structures. This makes it particularly suitable for DNA sequences, which lack clear word and sentence boundaries. BPE, originally developed as a compression algorithm, is widely used in natural language processing for tokenization. It generates a fixed-size vocabulary of variable-length tokens by learning character co-occurrence patterns. Table 2 summarizes the process of constructing a BPE vocabulary from a given corpus. Firstly, we initialize the vocabulary with all unique characters from the corpus. Subsequently, we iteratively identify the most frequent character segment, add it as a new word to the vocabulary, and update the corpus by replacing identical segments with the new word. This process is repeated until the vocabulary reaches the desired number of words.

**Table 2:** Example of the BPE vocabulary constructions.

**Iteration**	**Corpus**	**Vocabulary**
0	AACGCACTATATA	{A, T, C, G}
1	A A C G C A C TA TA TA	{A, T, C, G, TA}
2	A AC G C AC TA TA TA	{A, T, C, G, TA, AC}
3	A AC G C AC TA TA TA	……

The sequence module employs the embedding matrix initialized with a vocabulary size of 4096(2^12^). This matrix generates initial vector representations of DNA sequences after tokenization, enabling Inter-Chrom to fine-tune these embeddings for chromatin interaction prediction tasks.

During the initial sequence processing, a fixed-size vocabulary is constructed based on the co-occurrence frequency of words within the DNA sequences. To better leverage this information for subsequent model input, each sequence is filtered according to word length and frequency, retaining the top 500 words while preserving their original order. The iterative vocabulary construction reveals that longer words often capture unique sequence specificity and words of higher frequency epitomize more ubiquitous features. As a result, the subsequences from both dimensions contain distinct expressive features. Furthermore, to comprehensively capture sequence information, this procedure is applied to both forward and reverse orientations. Ultimately, each DNA sample, initially containing two sequences, is transformed into eight distinct subsequences based on different word patterns from both orientations.

The Efficient Channel Attention (ECA) module is designed to enhance the performance of deep neural networks by capturing inter-channel dependencies through one-dimensional convolution. This approach simplifies traditional attention mechanisms, eliminating the need for complex dimensionality reduction and enhancement steps, resulting in an efficient and lightweight model. The ECA module begins with global average pooling (GAP) to extract statistical information across channels. A one-dimensional convolutional kernel with an adaptive size is then applied to calculate the weights of each channel. These weights are transformed into attention coefficients using the Sigmoid activation function and then multiplied with the original feature maps to highlight important features while suppressing irrelevant ones. The kernel size k for convolution is calculated as follows:


(1) \begin{equation*} \mathrm{k}={\left|\frac{\log_2C}{\gamma }+\frac{b}{\gamma}\right|}_{odd}\end{equation*}

where C is the number of input channels, and γ and b are hyperparameters, typically set to 2 and 1, respectively. The value of k is rounded down to the nearest odd number to ensure compatibility with convolution operations. The attention mechanism of the ECA module is expressed as follows:


(2) \begin{equation*} {\displaystyle \begin{array}{c} Attention(F)=\sigma \left( Conv1{D}_k\left( GAP(F)\right)\right)\bigodot F\end{array}} \end{equation*}

where F represents the input feature, GAP denotes the global average pooling, Conv1D_k_ refers to the one-dimensional convolution with kernel size k, σ is the Sigmoid activation function, and ⊙ represents element-wise multiplication.

The ECA module is highly regarded for its computational efficiency, ability to preserve information integrity, adaptability in kernel size, and seamless integration with existing CNN architectures.

For each sample, the four distinct subsequences derived from sequence 1 undergo matrix transformation to produce embedded representations, which are then processed through independent ECA modules to obtain one-dimensional feature vectors. These vectors are merged with the corresponding feature vectors from sequence 2, processed similarly, and passed through a dense layer. The results are combined with outputs from other channels and processed through another dense layer.

The input to the genomic module undergoes batch normalization to accelerate network training, enhance generalization, and mitigate issues like vanishing or exploding gradients. The normalized data are processed through dense layers with activation functions and integrated with the sequence module output. To reduce overfitting, a dropout layer is applied before the final dense layer.

The model’s final predictions are generated through a sigmoid activation function, producing probabilities for classification. A threshold of 0.5 is employed: probabilities above 0.5 indicate positive interactions, while lower probabilities signify negative interactions. We use confusion matrix to further process the prediction results (Supplementary Table S1).

### Training procedure

To address the complex dependencies inherent in chromatin interaction data, we adopted a strategy to balance the independence and biological significance of signals. Using the updated dataset from targetfinder2, we implemented a chromosome-splitting method during cross-validation to eliminate dependencies between training and testing samples. This approach ensures that all samples from the same chromosome are confined to either the training or the testing set, effectively minimizing data leakage.

To handle class imbalance in the training set, we developed a specialized training strategy, as illustrated in Fig. 1(c). We divided the negative samples into multiple subsets based on the ratio of negative to positive samples. For each subset, along with all positive samples, an individual sub-model was trained. To mitigate overfitting, we split the training data into training and validation subsets at a 9:1 ratio and applied an early stopping mechanism during sub-model training.

After training all sub-models, we applied each one individually to predict outcomes for the test set. The final predictions were obtained by averaging the outputs from all sub-models. This ensemble method enhances the model’s ability to learn from minority class data, improving prediction accuracy and robustness. Meanwhile, we used grid search to determine the optimal parameters (detailed information in Supplementary Table S8).

### Evaluation metrics

To avoid overfitting caused by overlapping genomic regions between samples, we split the training and test sets by chromosomes rather than random assignment. Additionally, ten-fold cross-validation was employed for a comprehensive evaluation of model performance. The balanced test set, created through random undersampling, was evaluated using four metrics, including the Area Under the Precision-Recall Curve (AUPRC), Accuracy (ACC), Matthews Correlation Coefficient (MCC), and the F1 score. In general, higher values of these metrics reflect better model performance.

### Matching motifs

To identify the motifs in our dataset, we utilized known sequence motifs from the HOCOMOCO Human v11 [38] database. The Position Weight Matrix (PWM) of each motif was used to perform sliding window matches against the sequences in our dataset. For a motif of length L_m_, the PWM is represented as a matrix with L_m_ rows and four columns, corresponding to the nucleotide bases (A, C, G, and T). During the matching process, the sequence segments of length L_s_ were divided into a series of subsequences of length L_m_ with a step size of 1. For each subsequence, L_s_ - L_m_ + 1 matches were computed by summing the PWM scores for each base in the subsequence. This total score was then compared to a preset threshold to determine whether the subsequence matched the motif. The matching score for a subsequence is calculated as follows:


(3) \begin{equation*} {\displaystyle \begin{array}{c}Q=\sum\limits_{i=0}^{l_m-1}{PWM}_{ij}\end{array}} \end{equation*}

where j represents the four nucleotide bases (A = 0, C = 1, G = 2, T = 3), and Q denotes the matching score of the subsequence. A preset P-value threshold was assigned to each motif. If a subsequence’s matching score Q exceeded this threshold, the fragment was classified as a match for that motif.

Calculating the importance scores of motifs.

We propose a novel approach for calculating the importance scores of motifs, which accounts for several factors, including the motif length, average occurrence across samples, overall frequency among all motifs, and the change in performance metrics. The scoring formula is defined as follows:


(4) \begin{equation*} {\displaystyle \begin{array}{c}{Score}_m=\frac{\triangle_m}{C_m}\left[\alpha{f}_m{\log}_2\alpha{f}_m+1\right]\end{array}} \end{equation*}

(5) \begin{equation*} {\displaystyle \begin{array}{c}{\triangle}_m=P-{P}_m^{\prime}\end{array}} \end{equation*}

(6) \begin{equation*} {\displaystyle \begin{array}{c}{C}_m=\frac{1}{NUM_s}\sum\limits_{i=1}^{NUM_s}{C}_i\end{array}} \end{equation*}

(7) \begin{equation*} {\displaystyle \begin{array}{c}C=\sum\limits_{j=0}^{NUM_M}{C}_{M\left[j\right]}\end{array}} \end{equation*}

(8) \begin{equation*} {\displaystyle \begin{array}{c}{f}_m=\frac{C_m{l}_m}{\sum_{j=0}^{NUM_M}{C}_{M\left[j\right]}{l}_{M\left[j\right]}}\end{array}} \end{equation*}

(9) \begin{equation*} {\displaystyle \begin{array}{c}\alpha =\frac{e}{NUM_M}\end{array}} \end{equation*}

where P represents the model’s prediction outcome on the original input data, and ${P}_m^{\prime }$ denotes the prediction outcome after removing information about motif m from the input, using the same trained model. M represents the set of all motifs, and m refers to a specific motif. ${C}_m$ indicates the average number of times motif m appears in a sample, ${l}_m$ refers to the length of motif m, and ${f}_m$ signifies the proportion of motif m relative to all motifs in a sample. ${NUM}_s$ denotes the total number of samples, and ${NUM}_M$ represents the total number of motif types (401 in this study). $e$ represents the natural constant, and $\alpha$ denotes the correction factor. In this study, we uniformly use the AUPRC metric to evaluate the model’s predictive performance across various input data configurations. In order to facilitate readers' understanding of each step of calculating the importance score mentioned above, we provide a specific example in Supplementary Table S2.

When employing computational mutagenesis techniques to identify motifs for modification through motif matching, a critical challenge arises: determining whether certain subsequence regions, in combination with other content, carry essential information. We can observe from Fig. 1(d) that low-frequency motifs pose a particular difficulty, as increasing their occurrences might disrupt more critical information in the sample, requiring suppression. Conversely, highly frequent motifs should not have their significance underestimated, necessitating increased weight in their importance scoring.

To address this, our formula incorporates the entropy component from information theory. In this context, a higher entropy value reflects greater uncertainty in the information, while a lower value indicates less uncertainty. This concept is widely applied in fields such as data compression and cryptography [39]. The original entropy calculation is defined as follows:


(10) \begin{equation*} {\displaystyle \begin{array}{c}H(X)=-\sum\limits_{i=1}^np\left({x}_i\right)\log p\left({x}_i\right)\end{array}} \end{equation*}

where $p\left({x}_i\right)$ represents the probability of the random variable $X$ taking the value ${x}_i$. When event probabilities are evenly distributed, entropy increases; conversely, a greater disparity in probabilities results in lower entropy.

However, when translating this concept to elements like motifs, the behavior of entropy is inversely related to our goals. We argue that motifs with frequencies differing significantly from the average should be assigned higher importance scores. To achieve this, we utilize the $p\log p$ component of the entropy calculation. By incorporating the correction factor α, we ensure that the score is minimized when the motif proportion ${f}_m$ equals $1/{NUM}_M$. Deviations from this proportion, whether higher or lower, increase the motif’s importance score.

## Results

### Performance comparisons with other sequence-based models

Recent studies have utilized sequence analysis to predict chromatin interactions. However, the superior performance of certain methods may stem from the high overlap between training and test sets due to random data segmentation.

**Figure 2:**
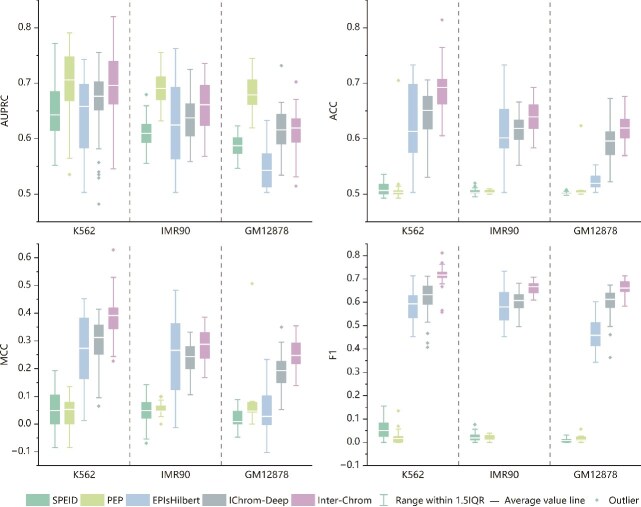
Performance comparison of sequence-based models in three cell lines. All results are evaluated 50 times and compared on the four evaluation metrics of AUPRC, ACC, MCC, and F1 score.

To ensure a fair comparison, we employed chromosome-splitting strategies and ten-fold cross-validation to evaluate the performance of SPEID, PEP, EPIsHilbert, IChrom-Deep, and Inter-Chrom, focusing solely on sequence data. Using a unified training strategy, we reassessed their effectiveness under consistent conditions (Fig. 2, detailed data in Supplementary Table S3). Our findings reveal that SPEID and PEP produced predictions close to random, aligning with observations by others., who suggested its performance may have been overestimated due to overfitting. While IChrom-Deep demonstrated its effectiveness by partially mitigating this issue, it still exhibited limitations in extracting sequence information effectively. In contrast, Inter-Chrom outperformed the other four methods across all four evaluation metrics and three cell lines. It demonstrated superior stability and robustness, highlighting its ability to extract and utilize sequence information more effectively than its counterparts.

### Performance evaluation across cell lines

The 3D structure of chromatin exhibits a degree of similarity across different cell types or states. This structural similarity is often reflected in the interaction patterns of specific chromatin regions, including the formation of loops or domains. Despite these overarching similarities, each cell type demonstrates unique chromatin accessibility and interaction patterns that directly influence its gene regulatory landscape. These cell-specific interactions play a pivotal role in the precise regulation of gene expression, enabling cells to perform distinct functions and maintain their identity. Therefore, investigating cell-specific chromatin interactions is crucial for revealing the molecular mechanisms underlying gene expression and epigenetic regulation.

**Figure 3:**
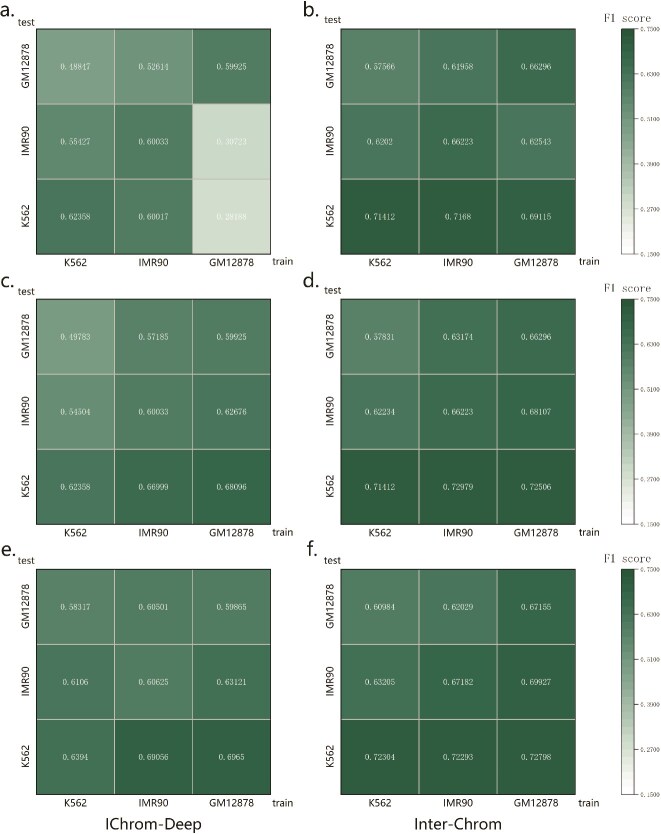
F1 score of IChrom-Deep and Inter-Chrom across cell lines. (a)-(f) show the results of the two methods under three different experimental conditions. (a) and (b) show results using the same number of samples for training and testing, strictly adhering to the chromosome-split strategy across cell lines. In this setup, group-based ten-fold cross-validation was performed on the training cell line, with groups defined by chromosome numbers. The chromosome numbers selected for testing in the ten-fold cross-validation were then matched to samples from the test cell line for final evaluation. (c) and (d) depict results using different numbers of samples for training and testing according to the dataset size for each cell line, while also using the chromosome-split strategy. (e) and (f) illustrate results using different numbers of samples for training and testing, evaluated through general ten-fold cross-validation without considering the grouping of chromosomes.

We evaluated the cross-cell line prediction capabilities of Inter-Chrom and compared them with IChrom-Deep. The two methods were assessed under three different experimental conditions. Figures 3(a) and3(b) depict results obtained using the same number of training and testing samples, strictly implementing the chromosome-split strategy across cell lines (detailed data in Supplementary Tables S4–S5).

In Fig. 3(a), the diagonal values are noticeably higher, indicating that IChrom-Deep struggles with cross-cell line prediction under these conditions. Detailed analysis reveals that prediction accuracy is strongly influenced by the chromosome, with particularly poor average F1 score when training on GM12878 data and testing on other cell lines, due to numerous invalid results. In contrast, Fig. 3(b) demonstrates that Inter-Chrom not only produced no invalid predictions across 50 trials under the same conditions, but its valid results were also significantly better than IChrom-Deep’s. These results of comparing Fig. 3(c) and (d) with Fig. 3(a) and (b) respectively confirm that larger training datasets lead to better model performance, consistent with prior findings [40, 41].

In Fig. 3(e) and (f), training and testing were conducted with varying sample sizes, using standard ten-fold cross-validation without chromosome grouping. The results were significantly higher than in the chromosome-split groups, underscoring the strong correlation between chromatin interactions and chromosomes. However, this exaggerated performance highlights the importance of employing chromosome-split strategies to prevent information leakage between training and testing sets, an issue inherent in standard ten-fold cross-validation for these tasks.

K562 cells are a highly proliferating cancer cell line, with typically more open chromatin and more open chromatin regions (OCRs), which may make it easier for models to capture long-range interactions when predicting chromatin interactions. In contrast, IMR90 is a normal fibroblast with a relatively compact chromatin state and relatively less OCR, which may limit the predictive performance of the model in certain regions. The histone modification patterns of different cell lines (such as H3K4me3, H3K27ac, etc.) show significant differences. For example, in K562 cells, the region labeled with H3K27ac is usually associated with active proliferation-related genes, while in GM12878, the region labeled with H3K27ac is more associated with immune-related genes. These differences may lead to different prediction preferences of the model for certain genomic regions in different cell lines [42]. The binding patterns of transcription factors vary significantly among different cell lines. For example, transcription factors from the MYC and ETS families are active in some cell lines, while transcription factors related to TP53 and fibroblast proliferation are more active in some cell lines [43]. The differences in CTCF binding sites and chromatin ring structure between GM12878 and K562 cell lines may lead to fluctuations in the performance of the model in cross-cell prediction [5].

### Performance comparison of modules with different input data combinations

**Figure 4:**
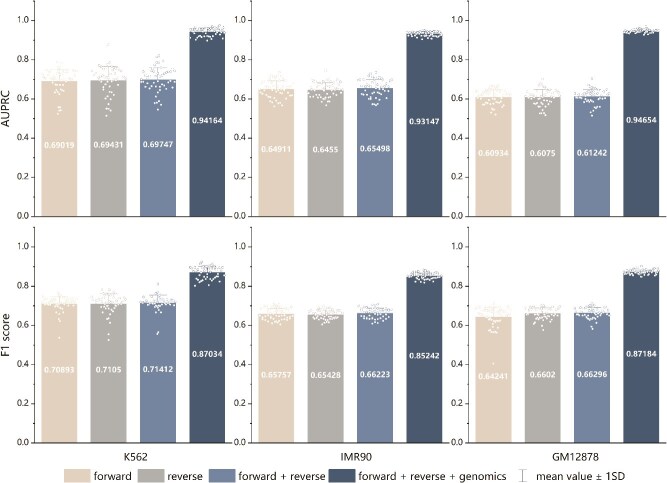
Average AUPRC and F1 score of models with different input data combinations across three datasets. The input combinations include ``forward'' (using only the forward DNA sequence), ``reverse'' (using only the reverse DNA sequence), and ``genomic'' (using all including genomic features).

We explored the impact of various input data combinations on model performance. Initially, forward and reverse DNA sequences were tested separately, yielding comparable results. When these sequences were combined into a full sequence module within the Inter-Chrom model, performance improved slightly (Fig. 4 and Supplementary Fig. S1, detailed data in Supplementary Table S6). This suggests that a single sequence input, supported by a robust feature processing module, can already achieve superior predictions compared to other methods. Nonetheless, adding feature inputs enhances performance further. Importantly, when computational resources or time are constrained, using only part of the sequence module (e.g. forward or reverse sequence) still delivers near-optimal results, providing a practical alternative for achieving satisfactory predictions.

Introducing genomic features as additional inputs significantly improved the model’s performance, emphasizing their crucial role in predicting chromatin interactions. This finding aligns with recent studies highlighting the importance of genomic features, suggesting that relying solely on sequence data for predicting chromatin interactions remains challenging. Notably, the integration of sequence data with genomic features significantly boosts the predictive performance of the model.

### Assessing the impact of DNA sequence mutations on model prediction

We investigated the effects of different DNA sequence mutation frequencies on model prediction performance, focusing on three mutation types: base substitutions (e.g. single nucleotide polymorphisms, SNPs), deletions, and insertions. Mutations in DNA sequences alter the input base sequence, with all three types affecting predictions of chromatin interactions, posing challenges to model adaptability. Notably, deletions and insertions impact not only sequence content but also sequence length.

To assess the model’s robustness to DNA sequence variations, we conducted experiments using the Inter-Chrom model with sequence-only inputs. For comparison, we also evaluated IChrom-Deep, which uses a fixed input length of 5000 bp. Due to this restriction, only base substitutions were tested for IChrom-Deep at mutation rates of 1%, 2%, 5%, and 10%. For Inter-Chrom, two groups were established: one testing base substitutions alone (allowing direct comparison with IChrom-Deep) and the other incorporating deletions and insertions, tested at the same mutation rates.

Prior studies indicate significant variability in mutation frequencies, with base substitutions being the most common. In contrast, deletions and insertions occur less frequently but can exert a profound impact due to the loss or addition of multiple nucleotides. Mutation incidence varies across genome regions and species, with deletions and insertions sometimes appearing at rates up to 40 times lower than point mutations [44].

**Figure 5:**
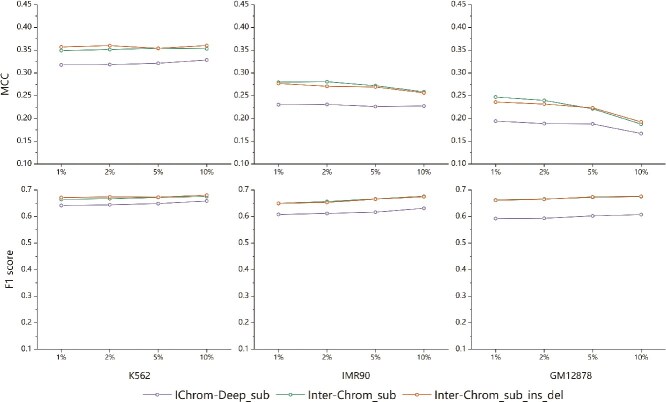
The variations in the average values of MCC and F1 score for both IChrom-deep and inter-Chrom on three main mutation types across three datasets at different rates. The three types of mutations include base substitution(sub), insertion(ins), and deletion(del).

To simplify the experimental design and more clearly observe differences, we set the perturbation frequency significantly higher than the natural mutation rate. Based on data from several studies [45], we established a simplified occurrence probability ratio of the three types of DNA mutations (point mutations, deletions, and insertions) at 10:1:1, making base substitutions an order of magnitude more frequent than the other mutation types. As illustrated by the two evaluation metrics presented in Fig. 5 (the other two evaluation indicators are shown in Supplementary Fig. S2 and detailed data in Supplementary Table S7), the predicted results across the four mutation frequencies exhibit similar trends among the experimental groups, with both Inter-Chrom groups outperforming IChrom-Deep. This indicates that, even with data perturbation, Inter-Chrom, which relies solely on sequence input, maintains an advantage in predicting chromatin interactions.

### Evaluating the importance of motifs in DNA sequences

In genomic research, transcription factors (TFs) bind to specific DNA sequences to regulate gene expression, playing a pivotal role in gene transcription [46]. Variations in these TF binding sites have also been associated with increased risks for various complex diseases [47]. Recent studies reveal that TFs often exhibit similar binding patterns within promoter and enhancer regions [48–50].

Accurate prediction of chromatin interactions relies on the model’s ability to identify key DNA sequence features that determine enhancer-promoter interactions (EPIs). However, current deep learning models fail to explicitly encode the features used for prediction, and the weights within deep neural networks do not necessarily correlate with feature importance.

In this study, we investigated how motif variations within input sequences affect prediction outcomes. We utilized known motifs from the HOCOMOCO Human v11 database and extracted relevant subsequence fragments using the matching method described in the Methods section. These fragments were then replaced with random noise, effectively erasing motif information from each sequence. This approach provides a precise evaluation of sequence changes on prediction results at the nucleotide level. While traversing each sequence, we collected occurrence counts for all motifs to facilitate subsequent importance score calculations. In the SPEID framework, motif feature importance is assessed by measuring the average drop in predictive performance. This is calculated by dividing the change in metric associated with each motif by its average occurrence across samples, thereby determining the unit influence of each motif on prediction results. However, this method does not account for the relative frequency of each motif compared to all motif occurrences within a sequence, which can impact its significance. To address this, we introduce a novel approach for evaluating motif importance, detailed in the Method section, which incorporates motif frequency differences into the assessment.

In the SPEID framework, a 20-bp window centered on the matching position is used to prevent bias towards longer motifs after identifying the motif location. For matches near the sequence ends, the last 20-bp of the sequence is modified. However, this approach unintentionally modifies more information from the original sequence data, especially for shorter motifs, making it ineffective at preventing bias towards longer motifs. Therefore, in our study, we only modify the matched regions when erasing motif information, and address other potentially confounding factors from a distinct perspective.

**Figure 6:**
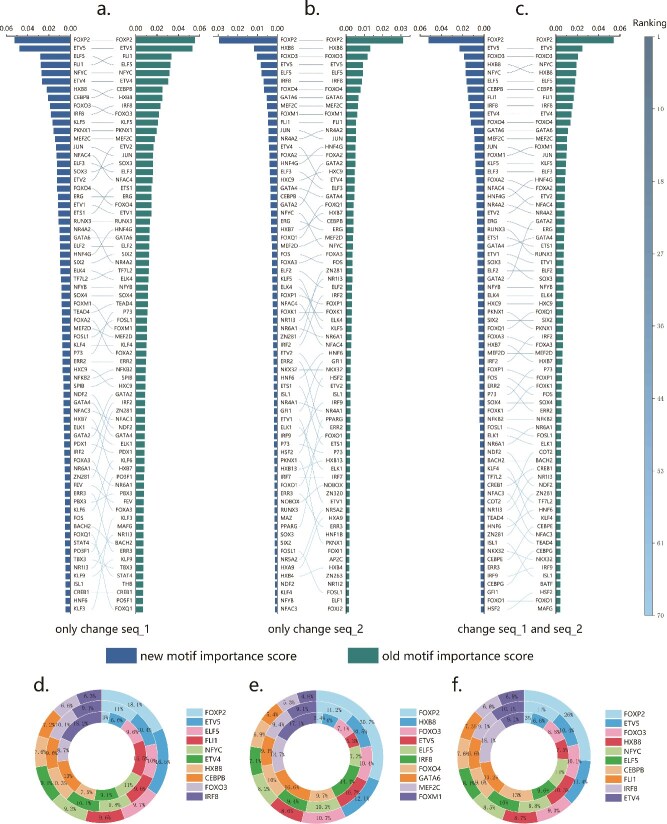
Motif importance ranking in the K562 cell line dataset using the new motif importance calculation method. (a)–(c) illustrate the differences in motif importance ranking between the new method proposed in this study and the traditional method. (a) represents the ranking of motif importance in seq_1 based on the changes in indicators when only seq_1 is modified. (b) shows the ranking of motif importance in seq_2 using the same methodology. (c) considers the overall situation in both sequences. (d)–(f) compare the importance scores of the top 10 motifs from the three experimental setups, alongside their respective motif proportions and correction factor sizes.

In our experiments, we utilized the Inter-Chrom model, which relies solely on sequence data as input. Initially, we trained the model on unmodified DNA sequences. Subsequently, we made predictions using sequences with specific motif information removed, resulting in 401 different changes in the result, each corresponding to the alteration of one motif. The experiments involved three conditions: in the first two conditions, we applied modifications to sequences from each chromatin bin in the interacting pair (seq_1 and seq_2) individually. In the third condition, we simultaneously modified both sequences based on a specified motif. Using the K562 cell line, as shown in Fig. 6(a), we compared motif importance scores generated by our method with those obtained using the SPEID scoring method. The information of cell lines IMR90 and GM12878 is shown in Supplementary Fig. S3 and Supplementary Fig. S4, respectively.

Figure 6(d), (e), and (f) present another representation of the results from the three experimental conditions using the K562 cell line. In these figures, we calculated three values for each motif: the first value (P) is the proportion of the motif, determined by multiplying its average occurrence by its length and then dividing by the total motif length; the second value (F) is the correction factor, which is calculated using the ratio P; and the third value (S) is the final importance score corresponding to the motif. These three values are shown for the top ten motifs with the highest importance scores in a circular pattern, with P in the center, F around it, and S in the outermost layer. By comparing the changes in proportions for the same motif, we observed that when a motif’s proportion is either small or large, the correction factor can increase, with fluctuations around 1.

Supplementary Fig. S5 shows the ranking of importance scores for 401 motifs obtained by two calculation methods before and after adding correction factors. Supplementary Fig. S5(a) reveals that the most important motifs tend to be concentrated in the smallest proportion, while the motif with the lowest importance score, located at the last element of the circular ring, has the highest proportion. This includes some widely studied motifs, such as the SP1 motif, which plays an important role in gene expression regulation, signaling pathway, and programmed cell death [51]. This observation suggests that calculating motif importance solely based on the difference in indicator values divided by motif frequency has its limitations. The importance ranking after the use of correction factors in Supplementary Fig. S5(b) not only makes the motifs with small proportions and high importance scores more concentrated, but also improves the ranking of motifs with a frequency much greater than $1/{NUM}_M$. The consistency between the top 10 motifs in Fig. 6(d), (e), and (f), and the global ranking in Supplementary Fig. S5 supports this finding. The dark blue thin ring in Supplementary Fig. S5(b) highlights a significant difference between the sum of the scores (S) and the proportion values (P), with 62.4% of the total importance scores of 401 motifs corresponding to only 6.7% of the motif occurrences. This result further underscores the necessity of incorporating motif proportion into our formula design, helping to prevent motifs that appear frequently from being overestimated in importance due to excessive interference with other potential motifs.

### Discussion on the influence of important motifs

**Figure 7:**
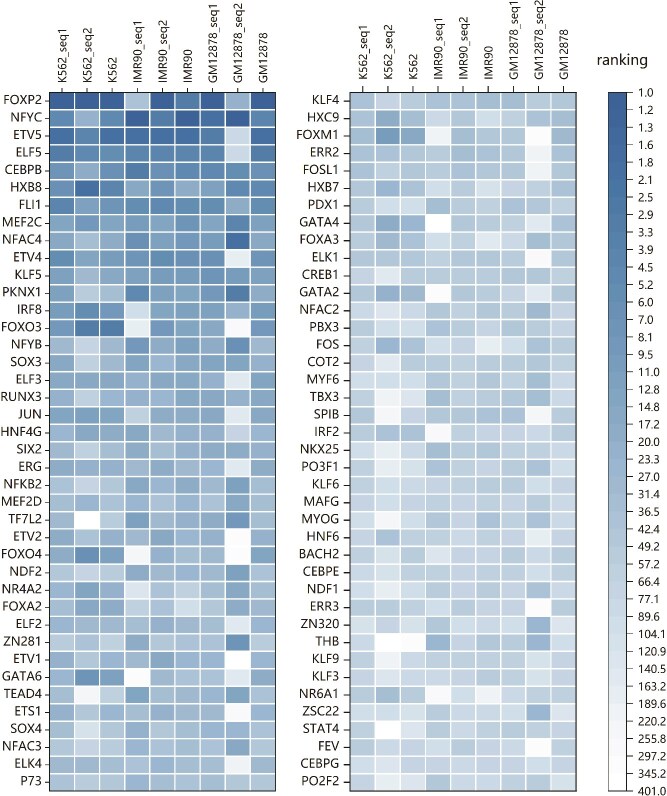
The performance of the top 20% motifs across different cell lines. This figure illustrates the performance of the top 20% motifs, ranked by importance scores, across different cell lines. For each cell line, three cases are analyzed: (i) importance scores of motifs in seq_1 based on changes in indicator values when only seq_1 is altered, (ii) importance scores when considering changes in seq_2, and (iii) importance scores when both sequences (seq_1 and seq_2) are modified simultaneously.

After ranking motif importance scores, we analyzed the top 20% of motifs to evaluate their performance across different cell lines, as shown in Fig. 7. The results reveal that, while most motifs demonstrate similar overall performance, some motifs display distinct behaviors in specific cell lines or chromatin bins. Using our enhanced calculation and ranking method, we identified important motifs that align with findings reported in numerous previous studies.

CEBPB is known to influence liver regeneration by regulating the expression of various genes. It also modulates inflammatory responses through cytokine gene regulation. Notably, CEBPB enhances the stem cell characteristics of cancer cells by upregulating genes related to epithelial-mesenchymal transition, thereby accelerating breast cancer progression [52–54]. NF-Y consists of three subunits (NFYA, NFYB, and NFYC) that work synergistically to bind specifically to the CCAAT motif in DNA, regulating the transcription of numerous genes [55–58].

FLI1, a transcription factor in the ETS family, plays crucial roles in hematopoietic system development, angiogenesis, and cancer [59–65]. Additionally, FLI1 collaborates with other transcription factors to regulate the expression of multiple developmental genes by mediating long-range chromatin interactions. FOXP2 is a transcription factor involved in neuronal development and function. It regulates gene expression through chromatin remodeling and transcriptional repression, modulating chromatin accessibility. Studies have revealed that FOXP2 also influences gene expression in neurons through long-range chromatin interactions, elucidating its role in maintaining genome structure and gene expression patterns [66–68].

MEF2C affects heart structure and function by regulating heart-specific genes and can also interact with chromatin-modifying enzymes [69, 70]. FOXO3 regulates cellular responses to oxidative stress by activating antioxidant genes (such as CAT and SOD2), contributing to stress resistance, delayed aging, and disease prevention [71–73]. ETV4 and ETV5, members of the ETS family, are crucial in cancer biology. ETV4 is also a downstream target of HER2, advancing breast cancer progression through enhanced proliferation and migration [74–77]. Similarly, ETV5 contributes to processes such as cell proliferation, differentiation, and tissue repair, making it a key factor in cancer progression, embryonic development, and tissue repair [78–80].

In addition to these well-studied transcription factors, our analysis highlighted several motifs with high rankings but limited research. For instance, HXB8 and HXC9, members of the HOX gene family, play a key role in development but are relatively understudied compared to other well-known HOX genes [81]. PKNX1, a transcription factor within the MECOM gene family, is another understudied motif with potential roles in neural development, hematopoiesis, and cancer [82]. Similarly, the functions and regulatory mechanisms of NDF2 have not been extensively studied, though existing literature suggests potential roles in cellular regulation and signaling pathways. These motifs may gain further validation for their importance in these fields in future studies.

## Discussion and conclusion

In this study, we proposed a novel deep learning model, Inter-Chrom, which leverages dynamic tokenization and DNABERT’s word embedding to predict chromatin interactions. The experimental results using only DNA sequence data as input showed that Inter-Chrom outperformed the compared methods across three cell line datasets, indicating the effectiveness of the sequence feature extraction module. Even under constrained computational resources, employing the smallest independent module of Inter-Chrom yielded results that were near-optimal and superior to other methods. These findings reinforce the robustness and versatility of the model. In cross-cell line validation, Inter-Chrom performed better than IChrom-Deep, and the similar fluctuations observed in several comparative experiments between the two methods further demonstrate that the similarity in chromatin interactions between the specified two cell lines obtained by the two methods is consistent. Another advantage of Inter-Chrom compared to other methods is its tolerance to changes in input data. The perturbation experiment includes three situations: base substitution, deletion mutation, and insertion mutation, the latter two of which will change the length of the input DNA sequence. Because IChrom-Deep can only receive sequences of fixed input length, the experiments related to it only include a few perturbation frequencies of base substitutions. The analysis of experimental results shows that the results under four perturbation frequencies in both groups of Inter-Chrom are superior to IChrom-Deep, which fully demonstrates the strong robustness of Inter-Chrom.

To explore the relationship between sequence features and the function of motifs, we conducted experiments using in silico mutagenesis, systematically replacing specific motifs with random noise and analyzing the resulting changes in predictions. This approach enabled the computation of motif importance scores, revealing numerous motifs critical to chromatin interactions. Importantly, we also identified motifs with potential significance that have not been extensively studied, presenting promising directions for future research.

The motif importance scoring formula proposed in this study offers broad applicability beyond the 401 motifs analyzed here. By accounting for both the proportional presence and unit impact of motifs within a population, the formula is adaptable to scenarios involving different motif counts or other sequence elements, providing a flexible solution for future studies with similar challenges.

Inter-Chrom represents a significant advancement in chromatin interaction prediction, driven by its robust sequence feature extraction module. The use of BPE for tokenizing DNA sequences addresses limitations associated with traditional k-mer tokenization, improving computational efficiency through non-overlapping tokens. However, this process introduces challenges. Specifically, the iterative nature of BPE creates a vocabulary biased toward shorter subsequences, potentially fragmenting longer motifs crucial for chromatin interaction predictions. Consequently, these motifs may not participate as independent units, leading to some loss of information. This trade-off between tokenization efficiency and motif integrity may partially explain why Inter-Chrom’s reliance solely on sequence data does not yield further accuracy improvements. Revisiting this balance could offer a pathway to enhancing model performance.

The phenomenon of unexpected improvement in model performance with increasing perturbation rate in perturbation experiments is due to multiple mechanisms. Moderately increasing the disturbance rate can be seen as a regularization method, which suppresses overfitting by introducing diverse data features. Its principle is consistent with the PGD multi-step disturbance enhancement model in adversarial training. At the same time, perturbations expand the model's learning ability for data distribution, similar to how fine-tuning perturbations in diffusion models can enhance generation diversity and quality, indicating that perturbations help capture more comprehensive feature patterns. In addition, perturbations may optimize the training dynamics by breaking the local optimal guidance model to explore a better solution space, which is consistent with the design logic of random noise optimization algorithms. However, there is a threshold for disturbance rate, and exceeding it can lead to performance degradation, which needs to be balanced with the characteristics of the task. This phenomenon provides a new perspective for understanding model generalization and optimization.

The current research has confirmed the adequacy of DNA sequence information in predicting chromatin interactions, but in the future, integrating multimodal data can further break through the upper limit of model performance and reveal more refined regulatory mechanisms.

The 3D spatial information provided by Hi-C data can directly reflect chromatin folding patterns and complement sequence features. For example, the C. Origami model significantly improved the prediction accuracy of cell-type-specific chromatin conformation by integrating Hi-C data with DNA sequences and CTCF binding sites [83]. Similarly, future models can enhance their ability to model long-range interactions by introducing Hi-C contact matrices. The MIDAS [84] algorithm integrates single-cell multi-omics data through generative artificial intelligence, successfully solving the problems of batch effects and mode loss. In the future, we can learn from such methods and incorporate single-cell resolution data into the framework to capture dynamic regulatory processes. The soScope model significantly improves the resolution and data quality of tissue structure by integrating spatial omics data with high-resolution morphological images. This suggests that combining spatial information can help the model distinguish chromatin interaction patterns in different cellular microenvironments [85].

Although DNA sequences and basic genomic features provide important information for the model, their limitations cannot be ignored. On the one hand, current models rely on static genomic features, making it difficult to capture the dynamics of cell states. Chromatin interactions may dynamically change the cell cycle, differentiation, or external stimuli. Although the integration of single-cell multi-omics data can partially solve this problem, time series modeling methods still need to be developed. On the other hand, although sequence features can encode local binding sites, they lack 3D spatial context and are unable to directly capture the spatial folding pattern of chromatin. Future research should prioritize integrating diverse data sources to establish comprehensive global constraints, thereby enhancing model generalizability and task-specific efficacy.

In summary, this study underscores the challenges and opportunities in predicting chromatin interactions using sequence-based models. While Inter-Chrom achieves notable success, the field continues to grapple with extracting meaningful features from noisy sequence data. Addressing these challenges and refining feature extraction methodologies could pave the way for breakthroughs in understanding chromatin interactions and their regulatory mechanisms.

**Key Points**

- We propose a bidirectional DNA sequence generalization strategy, extracting top-k words based on length and frequency.
- We develop Inter-Chrom, a novel architecture combining dynamic tokenization (adapting to variable sequence contexts), DNABERT’s word embedding, and the Efficient Channel Attention module to identify chromatin interactions.
- We demonstrate that Inter-Chrom achieves state-of-the-art performance across multiple metrics and demonstrates strong resilience to sequence length variations and perturbations.
- We analyze individual and overlapping motif contributions and propose a novel motif importance scoring approach to resolve these complexities.

## Supplementary Material

Supplementary_Information_bbaf289

## Data Availability

The raw data utilized in this study, along with comprehensive details of the Inter-Chrom’s code, are available for access at https://github.com/HaoWuLab-Bioinformatics/Inter-Chrom.
