# Supplementary material for: A novel deep learning framework with dynamic tokenization for identifying chromatin interactions along with motif importance investigation
Source: Brief Bioinform. 2025 Jun 19;26(3):bbaf289. doi: 10.1093/bib/bbaf289 (PMC12204613; doi:10.1093/bib/bbaf289)
Supplement: Supplementary_Information_bbaf289 [file supplementary_information_bbaf289.docx]

**Supplementary Information**

**1. Model evaluation metrics**

To ensure a comprehensive evaluation of Inter-Chrom and enable fair comparisons with existing methods, we employed four performance metrics, including the Accuracy (ACC), Area Under the Precision-Recall Curve (AUPRC), Matthews Correlation Coefficient (MCC), and the F1 score.

**1.1 Confusion matrix**

The confusion matrix is a fundamental tool in machine learning for evaluating the performance of classification models. It provides a detailed comparison of the model’s predictions against the actual labels, offering valuable insights into its classification performance. Represented as a two-dimensional matrix, each row corresponds to the true class labels, while each column represents the predicted class labels. For the specific case of binary classification, we present the confusion matrix in Table S1:

**Table S1. The confusion matrix corresponding to the binary classification problem.**

|  | | Predicted classes | |
| --- | --- | --- | --- |
|  |  | Predicted positive | Predicted negative |
| True classes | Positive class | True positive  (TP) | False negative  (FN) |
|  | Negative class | False positive  (FP) | True negative  (TN) |

As seen from Table S1, the confusion matrix for binary classification problems comprises four key elements: (1) True Positive (TP): the number of positive samples correctly predicted as positive by the model; (2) False Negative (FN): the number of positive samples incorrectly predicted as negative; (3) False Positive (FP): the number of negative samples incorrectly predicted as positive; and (4) True Negative (TN): the number of negative samples correctly predicted as negative.

In multi-class classification problems, such as those involving four classes (A, B, C, and D), the problem can be decomposed into four binary classification tasks: (1) Class A samples are treated as positive, with all non-Class A samples (i.e., Classes B, C, and D) treated as negative; (2) Class B samples are treated as positive, with non-Class B samples (i.e., Classes A, C, and D) treated as negative; (3) Class C samples are treated as positive, with non-Class C samples (i.e., Classes A, B, and D) treated as negative; and (4) Class D samples are treated as positive, with non-Class D samples (i.e., Classes A, B, and C) treated as negative. This approach facilitates the evaluation of the model's ability to distinguish each class from the rest.

**1.2 Accuracy**

Accuracy (ACC) measures the proportion of correctly classified instances relative to the total number of samples in the dataset. In the evaluation of multi-class models, ACC corresponds to the values of micro-F1, micro-recall, and micro-precision. It is computed using the following formula:

$$\begin{aligned} Accuracy=\frac{The number of samples correctly predicted by the model}{The total number of samples}\#\left( 1 \right) \end{aligned}$$

While ACC is a reliable indicator of model performance for balanced datasets, it can be biased in cases of class imbalance, where certain classes significantly outnumber others. In such scenarios, a model may skew predictions toward the majority class, achieving high accuracy even if minority class samples are poorly predicted.

**1.3 Area Under the Precision-Recall Curve**

The Area Under the Precision-Recall Curve (AUPRC) is a key metric for assessing the performance of binary classification models, particularly in datasets with imbalanced class distributions. AUPRC is defined as the integral of precision with respect to recall:

$$\begin{aligned} AUPRC=\int_{0}^{1} Precision(r)dr\#\left( 2 \right) \end{aligned}$$

where $Precision(r)$ denotes the precision at a specific recall level $r$. Unlike the Area Under the ROC Curve (AUC-ROC), which can be misleading in imbalanced datasets, AUPRC emphasizes the model's ability to correctly classify positive instances, making it particularly valuable in domains like medical diagnostics or fraud detection. AUPRC is typically calculated using numerical integration techniques applied to precision-recall pairs obtained at various classification thresholds.

**1.4 Matthews Correlation Coefficient**

The Matthews Correlation Coefficient (MCC) is a robust metric for evaluating binary classification performance, especially in imbalanced datasets. It is defined as:

$$\begin{aligned} MCC=\frac{TP\times TN-FP\times FN}{\sqrt{(TP+FP)(TP+FN)(TN+FP)(TN+FN)}}\#\left( 3 \right) \end{aligned}$$

MCC values range from -1 to +1, where +1 indicates perfect classification, -1 denotes complete misclassification, and 0 corresponds to random guessing. By considering the balance between all elements of the confusion matrix (true positives, true negatives, false positives, and false negatives), MCC provides a comprehensive evaluation, making it suitable for scenarios with unequal costs for false positives and false negatives.

**1.5 F1 score**

The F1 score is a widely used metric in binary classification and information retrieval, offering a balanced measure of precision and recall. It is the harmonic mean of precision and recall, computed as follows:

$$\begin{aligned} Precision=\frac{TP}{TP+FP}\#\left( 4 \right) \end{aligned}$$

$$\begin{aligned} Recall=\frac{TP}{TP+FN}\#\left( 5 \right) \end{aligned}$$

$$\begin{aligned} F1=2\times\frac{Precision\times Recall}{Precision+Recall}\#\left( 6 \right) \end{aligned}$$

The F1 score ranges from 0 to 1, with higher values indicating better model performance. A score of 1 represents perfect precision and recall, while a score of 0 reflects the absence of either precision or recall. This metric is particularly useful when false positives and false negatives have similar costs, providing a balanced evaluation of model accuracy. However, it does not account for true negatives, limiting its applicability in cases where true negatives play a significant role.

**2. Supplementary Figures**

**
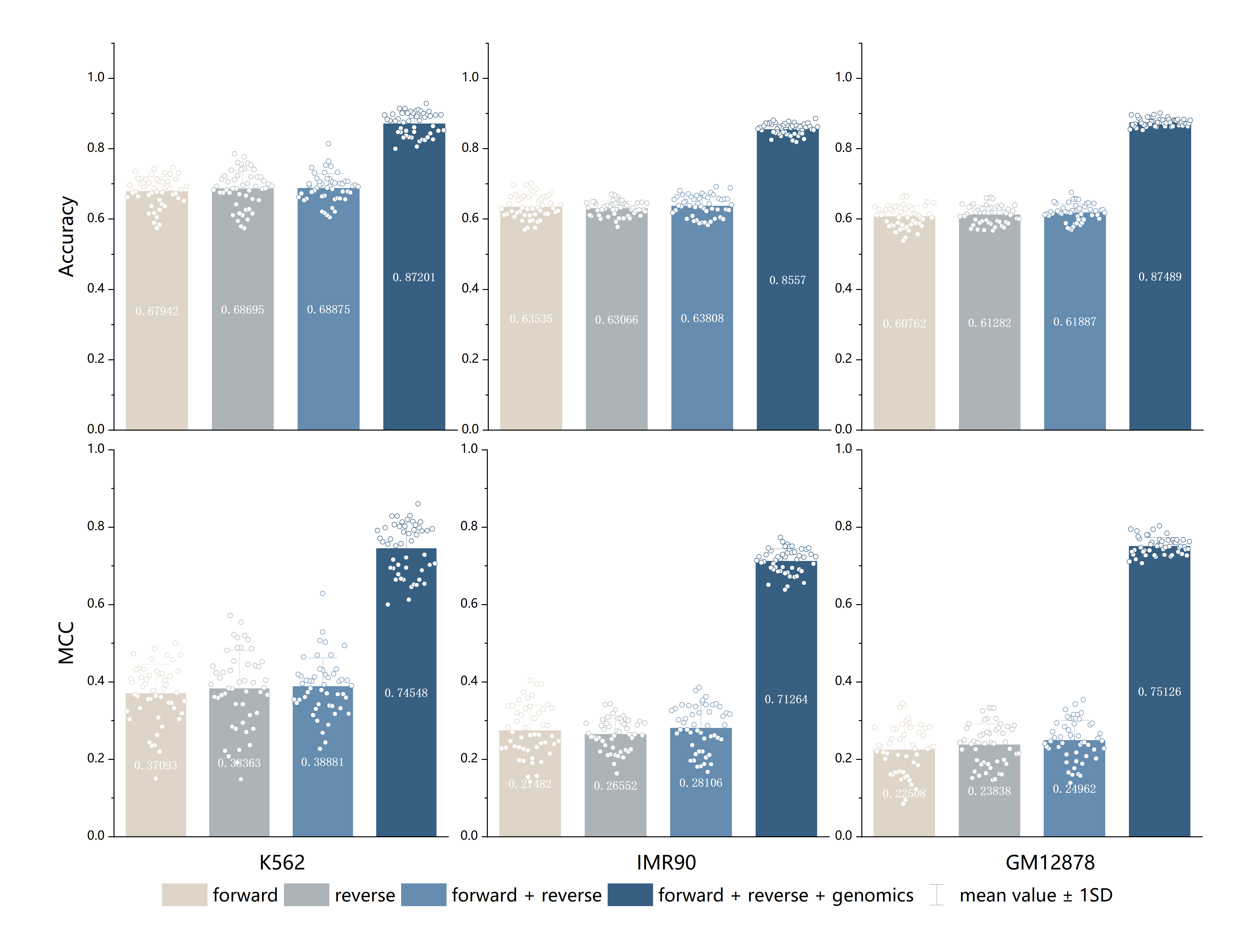
**

**Figure S1. The average values of Accuracy and MCC of models trained with different input data combinations across three datasets.** "forward" indicates the model's prediction results when only forward DNA sequence data is used as input. "reverse" represents the results when only reverse DNA sequence data is used, while "genomic" corresponds to the use of genomic features as input.

**
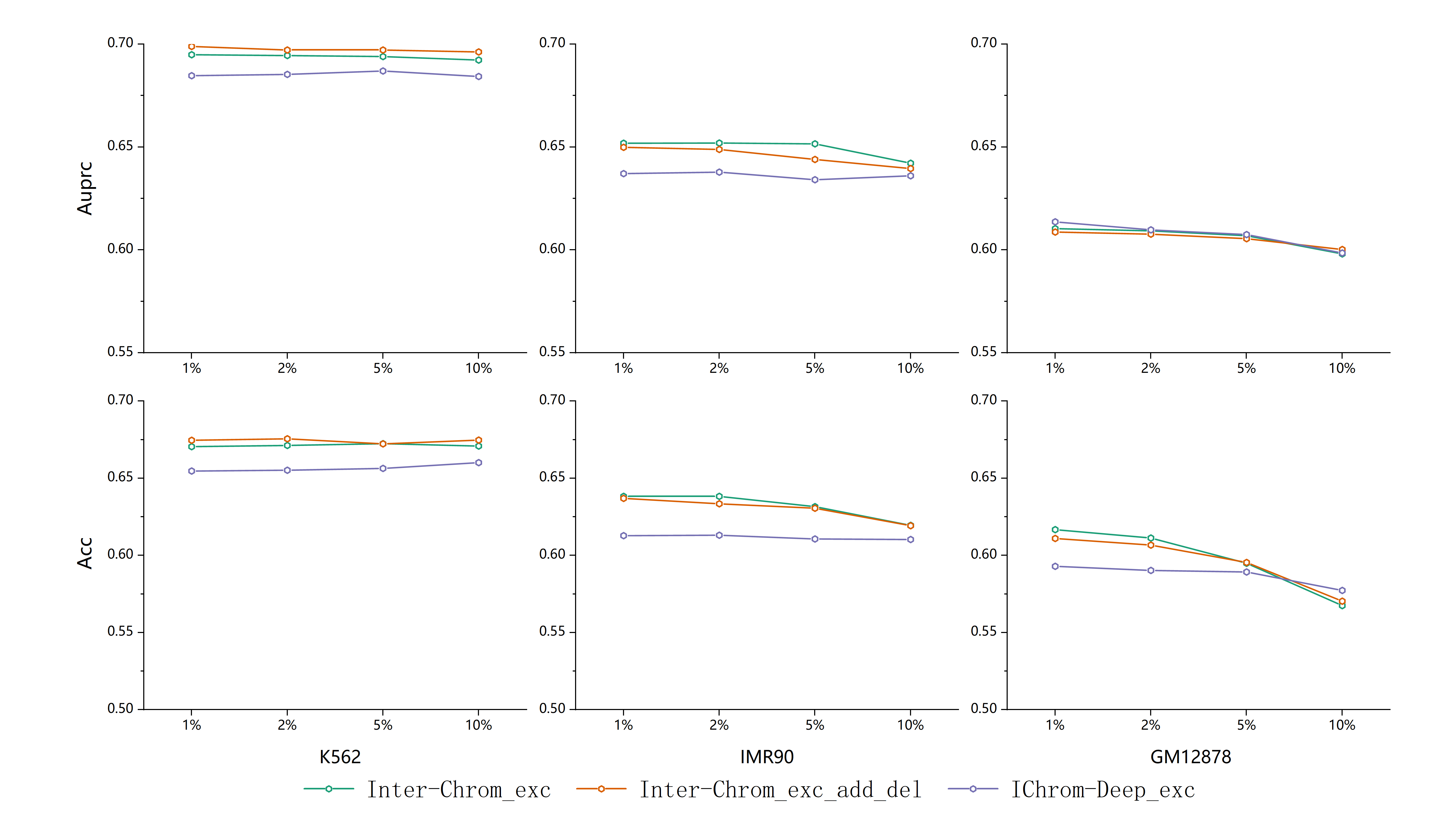
**

**Figure S2. Variations in the average values of AUPRC and ACC for both IChrom-Deep and Inter-Chrom across three primary mutation types at different rates, evaluated on three datasets.** The mutation types include base substitution(sub), insertion(ins), and deletion(del).

**
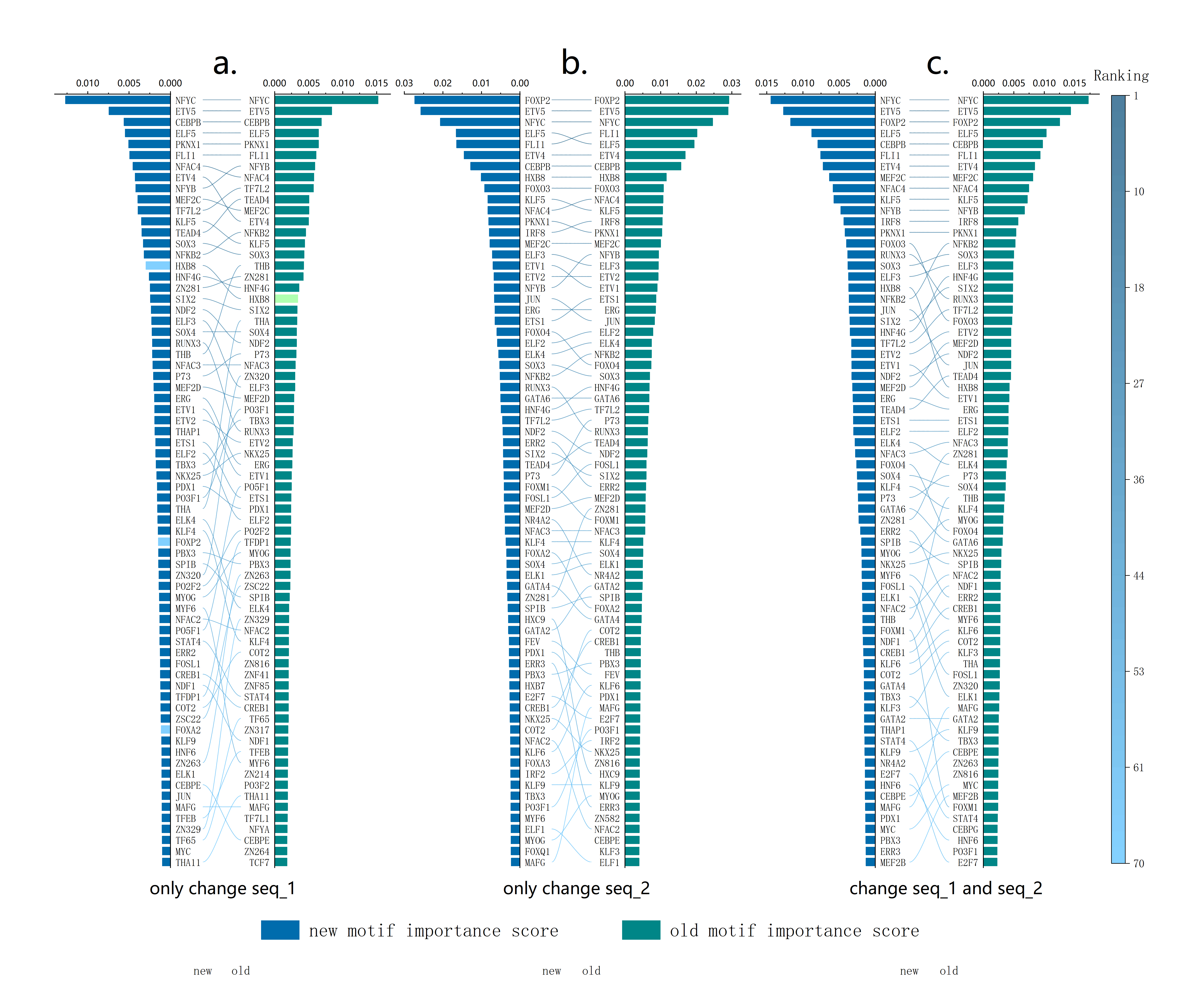
**

**Figure S3. Specific ranking of motifs in the IMR90 cell line dataset, derived using the newly proposed motif importance calculation method.** Figure (a)-(c) illustrate the changes in motif importance ranking between our method (the left) and the previous calculation method (the right). Figure (a) represents the ranking of motif importance in seq_1 based on the changes in indicators when only seq_1 is altered. Figure (b) shows the ranking of motif importance in seq_2 using the same method. Figure (c) considers the overall situation in both sequences. The light colored parts indicate negative scores.

**
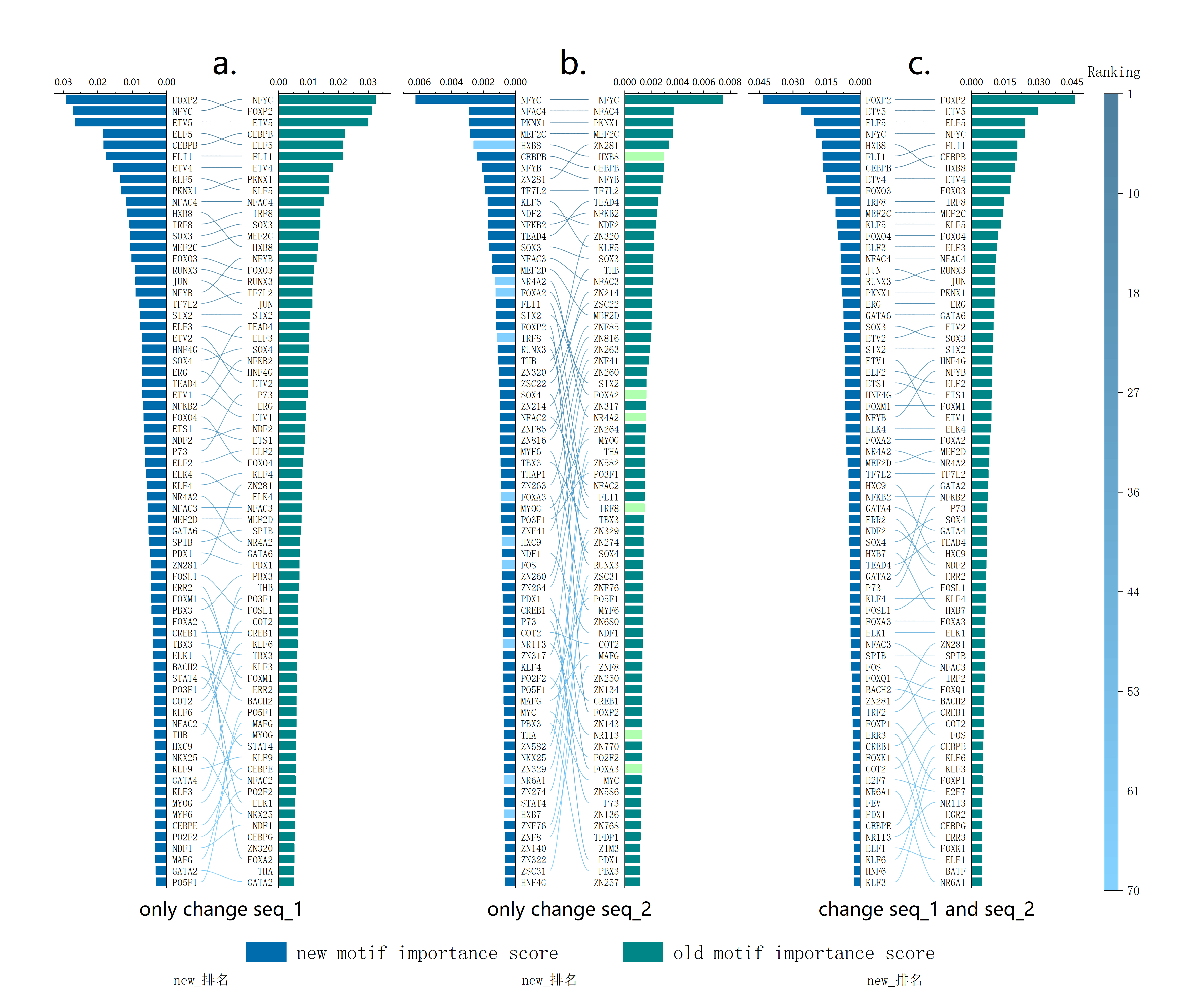
**

**Figure S4. Specific ranking of motifs in the GM12878 cell line dataset, obtained using the newly proposed motif importance calculation method.** Figure (a)-(c) illustrate the changes in motif importance ranking between our method (the left) and the previous calculation method (the right). Figure (a) represents the ranking of motif importance in seq_1 based on the changes in indicators when only seq_1 is altered. Figure (b) shows the ranking of motif importance in seq_2 using the same method. Figure (c) considers the overall situation in both sequences. The light colored parts indicate negative scores.

**
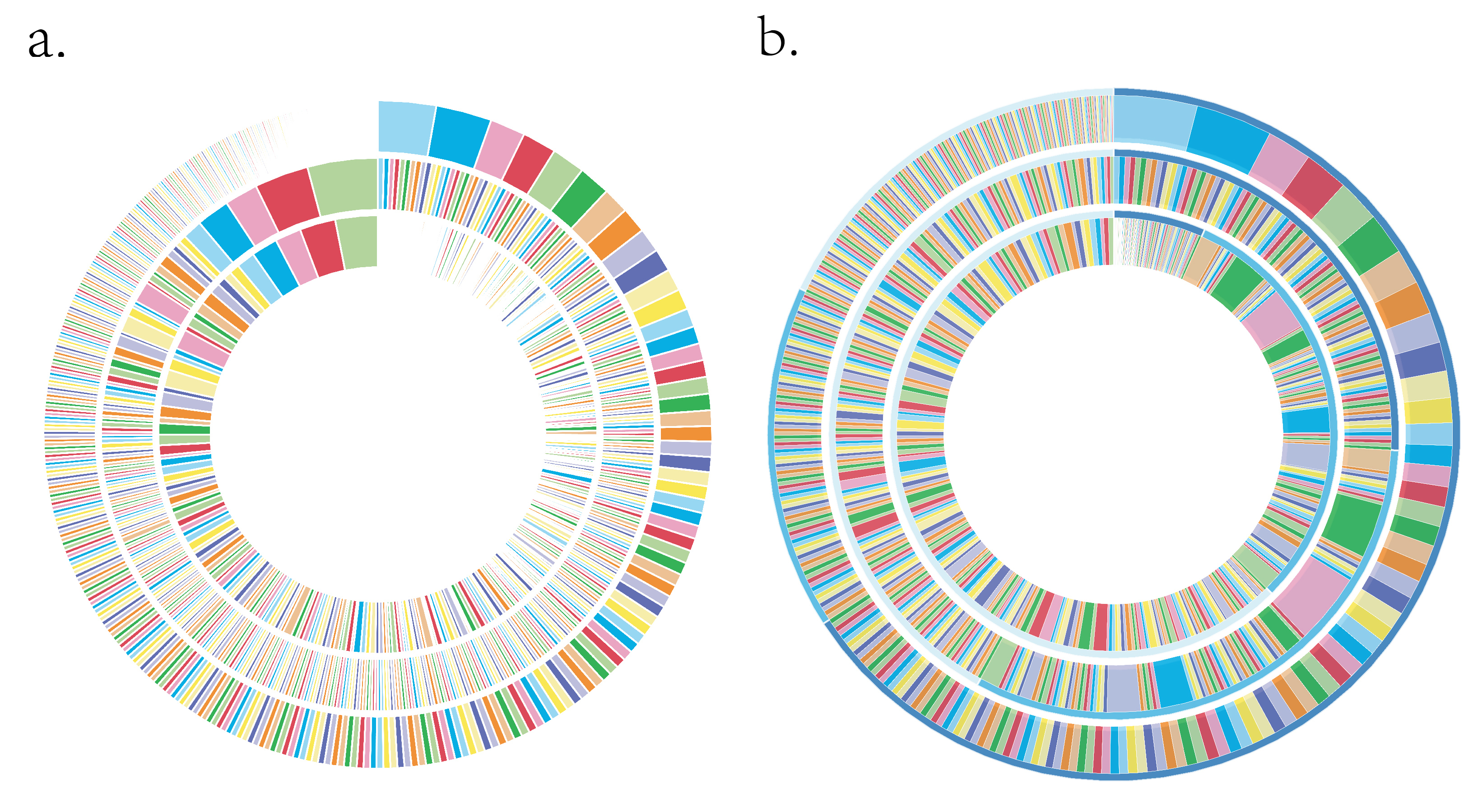
**

**Figure S5. Proportion of importance scores for 401 motifs in sequence_1 of the K562 cell line, calculated using two methods before and after the addition of correction factors.** (a) and (b) display comparisons of importance scores for all motifs, showing motif proportions and the sizes of corresponding correction factors. The calculation steps are as follows: P: The proportion of a motif is calculated by multiplying the average occurrences of the motif by its length and then dividing by the total length of all motifs. F: The correction factor, derived from the ratio of P. S: The final importance score corresponding to each motif. The top ten motifs with the highest importance scores are shown in a circular format, with the values of P, F, and S displayed from the inside out. A consistent pattern observed is that the most important motifs are concentrated within the smallest proportions.

**3. Supplementary Tables**

**3.1 Calculating the importance scores of motifs**

**Table S2.** A simple example of the specific steps of the formula for calculating the importance of motifs

| **Motif** | **A** | **B** | **C** | **D** | **E** |
| --- | --- | --- | --- | --- | --- |
| Length | 5 | 6 | 7 | 8 | 9 |
| Counts | 9 | 5 | 4 | 2 | 1 |
| Counts_Sum | 9+5+4+2+1=21 | | | | |
| C_m_ | 9/21 | 5/21 | 4/21 | 2/21 | 1/21 |
| Length* Counts | 45 | 30 | 28 | 16 | 9 |
| Length* Counts _Sum | 45+30+28+16+9=128 | | | | |
| f_m_ | 45/128 | 30/128 | 28/128 | 16/128 | 9/128 |
| $\triangle_{m}$ | P-P^’^_A_ | P-P^’^_B_ | P-P^’^_C_ | P-P^’^_D_ | P-P^’^_E_ |
| Score_m_(Take motif A for example) | $\frac{P-{P^{'}}_{A}}{9/21}*\left[ \frac{e}{401}*\frac{45}{128}\log_{2} (\frac{e}{401}*\frac{45}{128})+1 \right]$ | | | | |
| ${Score}_{m}=\frac{\triangle_{m}}{C_{m}}\left[ \alpha f_{m}\log_{2} \alpha f_{m}+1 \right]$ $\triangle_{m}=P-{P^{'}}_{m}$ $\alpha=\frac{e}{{NUM}_{M}}=\frac{e}{401}$ | | | | | |

**3.2 Experimental data**

**3.2.1 Performance comparisons with other sequence-based models**

**Table S3.** The performance of sequence-based models in three cell lines.

| **Cell line** | **Model** | **AUPRC** | **ACC** | **MCC** | **F1** |
| --- | --- | --- | --- | --- | --- |
| K562 | SPEID | 0.645233 | 0.509661 | 0.063356 | 0.055451 |
|  | PEP | 0.70064 | 0.50803 | 0.03646 | 0.02204 |
|  | EPIsHilbert | 0.640291 | 0.626919 | 0.262741 | 0.587106 |
|  | IChrom-Deep(sequence) | 0.666181 | 0.641453 | 0.293636 | 0.623576 |
|  | Inter-Chrom(sequence) | **0.697465** | **0.688748** | **0.388807** | **0.714117** |
| IMR90 | SPEID | 0.608451 | 0.504116 | 0.043343 | 0.022233 |
|  | PEP | 0.69059 | 0.50385 | 0.05146 | 0.01593 |
|  | EPIsHilbert | 0.626371 | 0.62191 | 0.24687 | 0.585253 |
|  | IChrom-Deep(sequence) | 0.638298 | 0.617055 | 0.240101 | 0.600334 |
|  | Inter-Chrom(sequence) | **0.654978** | **0.638083** | **0.281064** | **0.662228** |
| GM12878 | SPEID | 0.58765 | 0.50122 | 0.01984 | 0.00748 |
|  | PEP | 0.67969 | 0.50559 | 0.05911 | 0.01369 |
|  | EPIsHilbert | 0.548741 | 0.522939 | 0.050497 | 0.464748 |
|  | IChrom-Deep(sequence) | 0.614374 | 0.592828 | 0.191512 | 0.59925 |
|  | Inter-Chrom(sequence) | **0.612423** | **0.61887** | **0.249624** | **0.662961** |

**3.2.2 Performance evaluation across cell lines**

Condition 1: Train and test with the same number of samples, while strictly implementing the chromosome-splitting strategy across cell lines.

Condition 2: Train and test with different numbers of samples based on the size of each cell line dataset, while still applying the chromosome-splitting strategy.

Condition 3: Train and test with different numbers of samples and use general ten-fold cross-validation without considering chromosome grouping.

**Table S4.** The performance of Inter-Chrom across cell lines

| **condition** | **Train cell line** | **Test cell line** | **AUPRC** | **ACC** | **MCC** | **F1 score** |
| --- | --- | --- | --- | --- | --- | --- |
| 1 | K562 | K562 | 0.697465 | 0.688748 | 0.388807 | 0.714117 |
|  |  | IMR90 | 0.63958 | 0.62868 | 0.26128 | 0.6202 |
|  |  | GM12878 | 0.58662 | 0.5847 | 0.17133 | 0.57566 |
|  | IMR90 | K562 | 0.69912 | 0.67963 | 0.37762 | 0.7168 |
|  |  | IMR90 | 0.654978 | 0.638083 | 0.281064 | 0.662228 |
|  |  | GM12878 | 0.60765 | 0.60688 | 0.21907 | 0.61958 |
|  | GM12878 | K562 | 0.69255 | 0.66232 | 0.3513 | 0.69115 |
|  |  | IMR90 | 0.66236 | 0.62317 | 0.25898 | 0.62543 |
|  |  | GM12878 | 0.612423 | 0.61887 | 0.249624 | 0.662961 |
| 2 | K562 | K562 | 0.697465 | 0.688748 | 0.388807 | 0.714117 |
|  |  | IMR90 | 0.63732 | 0.62668 | 0.25694 | 0.62234 |
|  |  | GM12878 | 0.5844 | 0.58733 | 0.17718 | 0.57831 |
|  | IMR90 | K562 | 0.70308 | 0.69265 | 0.40359 | 0.72979 |
|  |  | IMR90 | 0.654978 | 0.638083 | 0.281064 | 0.662228 |
|  |  | GM12878 | 0.61089 | 0.61222 | 0.22933 | 0.63174 |
|  | GM12878 | K562 | 0.69162 | 0.67325 | 0.37483 | 0.72506 |
|  |  | IMR90 | 0.65401 | 0.63397 | 0.28226 | 0.68107 |
|  |  | GM12878 | 0.612423 | 0.61887 | 0.249624 | 0.662961 |
| 3 | K562 | K562 | 0.68535 | 0.69035 | 0.39291 | 0.72304 |
|  |  | IMR90 | 0.63611 | 0.62402 | 0.2561 | 0.63205 |
|  |  | GM12878 | 0.59476 | 0.59688 | 0.20126 | 0.60984 |
|  | IMR90 | K562 | 0.69473 | 0.68531 | 0.38835 | 0.72293 |
|  |  | IMR90 | 0.656 | 0.64633 | 0.29764 | 0.67182 |
|  |  | GM12878 | 0.61178 | 0.61211 | 0.23105 | 0.62029 |
|  | GM12878 | K562 | 0.70349 | 0.65637 | 0.36746 | 0.72798 |
|  |  | IMR90 | 0.67425 | 0.62903 | 0.29311 | 0.69927 |
|  |  | GM12878 | 0.61101 | 0.61523 | 0.24804 | 0.67155 |

**Table S5.** The performance of IChrom-Deep across cell lines

| **condition** | **Train cell line** | **Test cell line** | **AUPRC** | **ACC** | **MCC** | **F1 score** |
| --- | --- | --- | --- | --- | --- | --- |
| 1 | K562 | K562 | 0.666181 | 0.641453 | 0.293636 | 0.623576 |
|  |  | IMR90 | 0.6452 | 0.61507 | 0.24367 | 0.55427 |
|  |  | GM12878 | 0.59388 | 0.57222 | 0.15608 | 0.48847 |
|  | IMR90 | K562 | 0.66575 | 0.61635 | 0.25125 | 0.60017 |
|  |  | IMR90 | 0.638298 | 0.617055 | 0.240101 | 0.600334 |
|  |  | GM12878 | 0.60842 | 0.57367 | 0.15871 | 0.52614 |
|  | GM12878 | K562 | 0.65153 | 0.54255 | 0.10058 | 0.28188 |
|  |  | IMR90 | 0.6136 | 0.5386 | 0.09399 | 0.30723 |
|  |  | GM12878 | 0.614374 | 0.592828 | 0.191512 | 0.59925 |
| 2 | K562 | K562 | 0.666181 | 0.641453 | 0.293636 | 0.623576 |
|  |  | IMR90 | 0.62959 | 0.60578 | 0.22433 | 0.54504 |
|  |  | GM12878 | 0.58887 | 0.5719 | 0.15389 | 0.49783 |
|  | IMR90 | K562 | 0.67276 | 0.65296 | 0.31615 | 0.66999 |
|  |  | IMR90 | 0.638298 | 0.617055 | 0.240101 | 0.600334 |
|  |  | GM12878 | 0.60284 | 0.58693 | 0.17848 | 0.57185 |
|  | GM12878 | K562 | 0.69102 | 0.65716 | 0.32713 | 0.68096 |
|  |  | IMR90 | 0.6566 | 0.62056 | 0.24765 | 0.62676 |
|  |  | GM12878 | 0.614374 | 0.592828 | 0.191512 | 0.59925 |
| 3 | K562 | K562 | 0.67859 | 0.64917 | 0.30779 | 0.6394 |
|  |  | IMR90 | 0.63893 | 0.62216 | 0.25223 | 0.6106 |
|  |  | GM12878 | 0.59393 | 0.59058 | 0.18911 | 0.58317 |
|  | IMR90 | K562 | 0.68405 | 0.65253 | 0.32121 | 0.69056 |
|  |  | IMR90 | 0.64259 | 0.6168 | 0.2389 | 0.60625 |
|  |  | GM12878 | 0.60827 | 0.59358 | 0.19563 | 0.60501 |
|  | GM12878 | K562 | 0.67009 | 0.64615 | 0.31377 | 0.6965 |
|  |  | IMR90 | 0.64556 | 0.61402 | 0.23274 | 0.63121 |
|  |  | GM12878 | 0.61721 | 0.59942 | 0.20448 | 0.59865 |

**3.2.3 Performance comparison of modules with different input data combinations**

**Table S6.** Performance with different input data combinations across three datasets

| **Cell line** | **Model** | **AUPRC** | **ACC** | **MCC** | **F1** |
| --- | --- | --- | --- | --- | --- |
| K562 | forward | 0.69019 | 0.67942 | 0.37093 | 0.70893 |
|  | reverse | 0.69431 | 0.68695 | 0.38363 | 0.7105 |
|  | forward+reverse | 0.697465 | 0.688748 | 0.388807 | 0.714117 |
|  | all features | **0.94164** | **0.87201** | **0.74548** | **0.87034** |
| IMR90 | forward | 0.64911 | 0.63535 | 0.27482 | 0.65757 |
|  | reverse | 0.6455 | 0.63066 | 0.26552 | 0.65428 |
|  | forward+reverse | 0.654978 | 0.638083 | 0.281064 | 0.662228 |
|  | all features | **0.93147** | **0.8557** | **0.71264** | **0.85242** |
| GM12878 | forward | 0.60934 | 0.60762 | 0.22508 | 0.64241 |
|  | reverse | 0.6075 | 0.61282 | 0.23838 | 0.6602 |
|  | forward+reverse | 0.612423 | 0.61887 | 0.249624 | 0.662961 |
|  | all features | **0.94654** | **0.87489** | **0.75126** | **0.87184** |

**3.2.4 Assessing the impact of DNA sequence mutations on model prediction**

**Table S7.** The variations of score for both IChrom-Deep and Inter-Chrom on three main mutation types across three datasets at different rates.

|  | **Cell line** |  | **1%** | **2%** | **5%** | **10%** |
| --- | --- | --- | --- | --- | --- | --- |
| **MCC** | K562 | IChrom-Deep_sub | 0.31773 | 0.31845 | 0.32121 | 0.32847 |
|  |  | Inter-Chrom _sub | 0.34916 | 0.35149 | 0.35397 | 0.35311 |
|  |  | Inter-Chrom _sub_ins_del | 0.35713 | 0.35992 | 0.35421 | 0.36012 |
|  | IMR90 | IChrom-Deep_sub | 0.23026 | 0.23082 | 0.22618 | 0.22741 |
|  |  | Inter-Chrom _sub | 0.27972 | 0.2806 | 0.27206 | 0.25856 |
|  |  | Inter-Chrom _sub_ins_del | 0.27698 | 0.27067 | 0.26914 | 0.25617 |
|  | GM12878 | IChrom-Deep_sub | 0.19416 | 0.18864 | 0.18812 | 0.16686 |
|  |  | Inter-Chrom _sub | 0.2471 | 0.23965 | 0.22113 | 0.18762 |
|  |  | Inter-Chrom _sub_ins_del | 0.23613 | 0.23175 | 0.22334 | 0.19243 |
| **F1 score** | K562 | IChrom-Deep_sub | 0.64175 | 0.64429 | 0.64873 | 0.65875 |
|  |  | Inter-Chrom _sub | 0.6646 | 0.66727 | 0.67195 | 0.67534 |
|  |  | Inter-Chrom _sub_ins_del | 0.67153 | 0.67382 | 0.67282 | 0.68047 |
|  | IMR90 | IChrom-Deep_sub | 0.60799 | 0.61166 | 0.61644 | 0.63113 |
|  |  | Inter-Chrom _sub | 0.64963 | 0.65683 | 0.66655 | 0.67651 |
|  |  | Inter-Chrom _sub_ins_del | 0.64976 | 0.6532 | 0.6656 | 0.67478 |
|  | GM12878 | IChrom-Deep_sub | 0.59242 | 0.59366 | 0.60256 | 0.60745 |
|  |  | Inter-Chrom _sub | 0.66243 | 0.66612 | 0.67256 | 0.67515 |
|  |  | Inter-Chrom _sub_ins_del | 0.66129 | 0.66521 | 0.67404 | 0.67633 |

**3.2.5 Hyperparameter selection**

Patience refers to the maximum number of rounds in the early stopping strategy where the loss of the validation set is not decreasing.

lr: learning rate

**Table S8.** F1 score obtained by combining different hyperparameters

| **patience** | **batch_size** | **lr** | **F1 score** | **patience** | **batch_size** | **lr** | **F1 score** |
| --- | --- | --- | --- | --- | --- | --- | --- |
| **5** | 16 | 1e-5 | 0.6812 | **15** | 16 | 1e-5 | 0.6923 |
|  |  | 1e-4 | 0.6987 |  |  | 1e-4 | 0.7056 |
|  |  | 5e-4 | 0.6741 |  |  | 5e-4 | 0.6879 |
|  |  | 1e-3 | 0.6423 |  |  | 1e-3 | 0.6589 |
|  | 32 | 1e-5 | 0.6935 |  | 32 | 1e-5 | 0.7031 |
|  |  | 1e-4 | 0.7059 |  |  | 1e-4 | 0.7168 |
|  |  | 5e-4 | 0.7012 |  |  | 5e-4 | 0.7093 |
|  |  | 1e-3 | 0.6538 |  |  | 1e-3 | 0.6672 |
|  | 64 | 1e-5 | 0.6654 |  | 64 | 1e-5 | 0.6781 |
|  |  | 1e-4 | 0.6823 |  |  | 1e-4 | 0.6934 |
|  |  | 5e-4 | 0.6731 |  |  | 5e-4 | 0.6857 |
|  |  | 1e-3 | 0.6312 |  |  | 1e-3 | 0.6478 |
| **10** | 16 | 1e-5 | 0.6897 | **20** | 16 | 1e-5 | 0.6954 |
|  |  | 1e-4 | 0.7071 |  |  | 1e-4 | 0.7123 |
|  |  | 5e-4 | 0.6882 |  |  | 5e-4 | 0.6945 |
|  |  | 1e-3 | 0.6598 |  |  | 1e-3 | 0.6631 |
|  | 32 | 1e-5 | 0.7043 |  | 32 | 1e-5 | 0.7089 |
|  |  | 1e-4 | **0.7141** |  |  | 1e-4 | **0.7132** |
|  |  | 5e-4 | **0.7106** |  |  | 5e-4 | **0.7134** |
|  |  | 1e-3 | 0.6691 |  |  | 1e-3 | 0.6725 |
|  | 64 | 1e-5 | 0.6779 |  | 64 | 1e-5 | 0.6832 |
|  |  | 1e-4 | 0.6912 |  |  | 1e-4 | 0.6987 |
|  |  | 5e-4 | 0.6843 |  |  | 5e-4 | 0.6921 |
|  |  | 1e-3 | 0.6419 |  |  | 1e-3 | 0.6523 |
